# 

**Published:** 1918-07-13

**Authors:** 


### CONTENTS.

PAGE

**King George and Queen Mary** ... ... ... ... 315

**Institutional Medical Relief in London** ... 316

**Hospital and Institutional News** ... ... ... 317

Sir Francis Lloyd on Voluntary Effort—Hospital Ships Searched by Germans—The American Hospital near Southampton—An Infirmary Chaplain's "Esprit de Corps"—The Right to Introduce Visitors Without Permission—The Price of Taking Schools for Hospitals—The Fire at Cardiff—Improvements at Gloucester Royal Infirmary—The Bute Family and King Edward VII. Hospital, Cardiff—An Example to be Followed up at York—A Unanimous Resolution—A New Scale of Fees for Doctors—The Chance of a Lifetime for Medical Women—An American Unit for Palestine—Salaries for Vaccination Officers—A New Municipal Post—An Atlas for the Blind—Where to Keep the Milk—The Army Pharmaceutical Service: A Belated Concession—Increased Salary for Poor-Law Dispensers—The Amusements of Exile—This Week's Drug Market.

**The Great War** ... ... ... ... ... ... 321

X. Personal Cleanliness: Vermin.

**Temporary Artificial Legs** ... ... ... ... 323

As Made at the Pavilion General Hospital, Brighton.

**The "Llandovery Castle" Outrage** ... ... 325

Survivor's Story to the King.

**The New Clinic at Liverpool Royal Infirmary** 325

PAGE

**The Registration of Nurses** ... ... ... 326

By What Process Would Registration Contribute to the Prosperity of the British Nurse?

**The Matrons' and Sisters' Department** ... 327

Nurse Registration in America.

Round the Hospitals.

Queen Mary's Mother's Presentiment—The Murdered Matrons and Sisters—London Hospital Nurses—Libelling American Nurses—A Problem for Laundry Managers—Future Prospects of the V.A.D.

**London Baby Week** ... ... ... ... ... 331

Some Lessons of the Conference.

**War Charity and Relief** ... ... ... ... 332

Funds of the Past, Present, and Future.

**The College of Nursing in Scotland** ... ... 333

**London Hospital Nurses** ... ... ... ... 333

Questions in Parliament.

Viscount Knutsford's Reply in *The Times*.

**News from Australia** ... ... ... ... ... 334

**Parliament and the Profession** ... ... ... 334

**Matrons' and Sisters' Appointments** ... ... iv

**Personalia** ... ... ... ... ... ... ... iv

**The Editor's Letter-Box** ... ... ... ... iv

**Some Annual Meetings** ... ... ... ... iv

**The Institutional Worker** ... ... (Suppl.) 1

The Preservation of Fruit and Vegetables.

